# Avenanthramide C From Oats Possibly Exerts Anti‐Inflammatory Effects in Human Umbilical Vein Endothelial Cells

**DOI:** 10.1111/1750-3841.70841

**Published:** 2026-01-09

**Authors:** Hiroyuki Sasaki, Hirofumi Masutomi, Hajime Nagasawa, Yuma Matsumoto, Teruyuki Okuma, Tomoyuki Otsuka, Katsuyuki Ishihara, Yusuke Suzuki, Seiji Ueda

**Affiliations:** ^1^ Research and Development Division Calbee, Inc. Utsunomiya Tochigi Japan; ^2^ Division of Kidney Health and Aging, the Center for Integrated Kidney Research and Advance Shimane University Faculty of Medicine Izumo Shimane Japan; ^3^ Department of Nephrology Juntendo University Faculty of Medicine Tokyo Japan; ^4^ Division of Nephrology, Department of Internal Medicine, Faculty of Medicine Shimane University Izumo Shimane Japan

**Keywords:** avenanthramide C, HUVECs, indoxyl sulfate, polyphenol

## Abstract

Chronic kidney disease (CKD) is associated with inflammation and cardiovascular complications and is partly exacerbated by the uremic toxin indoxyl sulfate (IS). IS is known to activate the aryl hydrocarbon receptor (AhR) to promote vascular inflammation. On the other hand, avenanthramide C (Ave), an oat‐derived polyphenol, has antioxidative and anti‐inflammatory properties. Therefore, we investigated whether Ave can suppress IS‐induced inflammatory responses.

Analysis of serum from hemodialysis patients revealed a significant correlation between IS and interleukin‐6 (IL‐6) levels. In human umbilical vein endothelial cells (HUVECs), IS (≥50 µg/mL) increased IL‐6 secretion, while Ave (≥10 µM) suppressed this effect. Docking simulations and in vitro experiments suggested that Ave may interact with AhR and suppress IS‐induced expression of AhR target genes, including that of cytochrome P450 family 1 subfamily A member 1. High‐performance liquid chromatography verified the uptake of Ave by human umbilical vein endothelial cells. Additionally, the anti‐inflammatory effects of Ave were independent of the adrenergic α1 receptor, adenosine monophosphate‐activated protein kinase pathways, and protein kinase B pathways.

Ave suppresses IS‐induced inflammation, thereby reducing IL‐6 secretion in HUVECs. These findings suggest the potential of Ave as a dietary intervention for mitigating vascular inflammation in patients with CKD.

**Abbreviations**
AhR: Aryl hydrocarbon receptor
Akt: Protein kinase B
AMPK: Activated protein kinase
Ave: Avenanthramide C
CKD: Chronic kidney disease
DMP: Dorsomorphin
ELISA: Enzyme‐linked immunosorbent assay
HUVECs: Human umbilical vein endothelial cells
IL‐6: Interleukin‐6
IS: Indoxyl sulfate
NF‐κB: Nuclear factor‐kappa‐B
PI3K: Phosphoinositide 3‐kinase
TNF‐α: Tumor necrosis factor‐α

## 1 Introduction

The number of hemodialysis patients has surpassed 300,000, leading to its recognition as a significant public health concern in Japan (Hanafusa et al. 2024). Patients with chronic kidney disease (CKD) develop progressive atherosclerosis even before initiating hemodialysis, resulting in a significantly higher incidence and mortality rate due to cardiovascular diseases (Go et al. 2004). Among the various factors contributing to cardiovascular disease in patients with CKD, the uremic toxin IS is particularly notable. IS is a sulfated metabolite derived from dietary proteins. Upon protein intake, tryptophan is released through digestion and subsequently metabolized by the gut microbiota into indole. In the liver, indole is oxidized, and IS is synthesized via sulfotransferase family 1A member 1 (Hou et al. 2023; Yabuuchi et al. 2021). In individuals with kidney impairment, its elimination is compromised, leading to systemic accumulation in various organs, including the bloodstream, kidneys, lungs, and skeletal muscles. This accumulation exacerbates oxidative stress and triggers inflammatory pathways, ultimately contributing to vascular endothelial dysfunction and an increased risk of cardiovascular disease (Daenen et al. 2019). The mechanism by which IS induces inflammatory responses in vascular endothelial cells has been well characterized. IS is transported into cells via organic anion transporter 3, where it activates the aryl hydrocarbon receptor (AhR), leading to phosphorylation of the nuclear factor NF‐kappa‐B (NF‐κB) p65 subunit. This phosphorylation facilitates the translocation of the nuclear factor NF‐κB p65 subunit to the nucleus, where it modulates the expression of various inflammation‐related genes (Adelibieke et al. 2014). Given its role in promoting inflammation, reducing circulating IS levels is crucial. Although the oral adsorbent activated charcoal spherical adsorbent (AST‐120) is used in clinical practice to mitigate IS accumulation, its efficacy is often limited owing to poor patient adherence (Kee et al. 2021; Niwa 2017). In our previous study, we have demonstrated that a two‐month intervention with granola supplementation in hemodialysis patients led to a reduction in circulating IS levels. This decrease was accompanied by modifications in gut microbiota composition, improved bowel movement regularity, and lower blood pressure (Nagasawa et al. 2024; Nagasawa et al. 2021).

Oat consumption has been associated with a reduced risk of several chronic diseases, including cancer, type II diabetes, and cardiovascular disease (Erkkilä et al. 2005; Jacobs et al. 1998; Jensen et al. 2006; Liu et al. 2000a, 2000b; Liu et al. 1999). Among the bioactive compounds in oats, avenanthramide C (Ave), a polyphenol, has been reported to alleviate oxidative stress and inflammation and suppress obesity in mice (Zhang et al. 2020). The antioxidative properties of Ave are primarily attributed to its ability to scavenge reactive oxygen species by increasing glutathione levels (Chen et al. 2004; Liu et al. 2011; Yang et al. 2014). In a study involving adult women subjected to high‐intensity exercise, Ave intake significantly reduced inflammation by inhibiting NF‐κB activation, lowering circulating interleukin‐6 (IL‐6) levels, enhancing erythrocyte glutathione peroxidase activity, and increasing glutathione concentrations (Koenig et al. 2014; Koenig et al. 2016). These findings indicate that Ave may play a role in the attenuation of systemic inflammatory responses. One previously proposed mechanism has suggested that Ave exerts anti‐inflammatory effects by activating adenosine monophosphate‐activated protein kinase (AMPK) via the α1‐adrenergic receptor in the hippocampus of an Alzheimer's disease mouse model (Ramasamy et al. 2020). Additionally, other reports have indicated that Ave may suppress the phosphoinositide 3‐kinase (PI3K) signaling pathway in neurodegenerative diseases, leading to anti‐inflammatory effects (Wankhede et al. 2023). Despite these insights, no studies have examined the mechanism of action of Ave in the context of CKD or vascular endothelial cells. Therefore, the objective of this study was to investigate whether oat‐derived Ave inhibits the inflammatory response induced by IS and elucidate the underlying mechanisms.

## 2 Experimental Section

### 2.1 Human Experiments

The human experiments in this study were conducted using serum samples collected in a previous study (Nagasawa et al. 2024). Of the hemodialysis patients at the Izu Nagaoka Daiichi Clinic, 24 agreed to participate in this study. However, patients with malignancies, active inflammation, receiving steroid therapy, or with poor nutritional status (defined as a Geriatric Nutritional Risk Index <90) were excluded. All participants provided written informed consent prior to enrollment. The study protocol was approved by the Ethics Committee of Juntendo University (approval number: 17–247) and was conducted in accordance with the principles of the Declaration of Helsinki. This study was registered in the University Hospital Medical Information Network Clinical Trial Registry (registration number: UMIN000031666).

#### 2.1.1 Biochemical Parameter Measurements

Blood samples were obtained from the arterial hemodialysis line. The serum was then immediately frozen at −80 °C until analysis. Serum levels of IS, a representative gut‐derived uremic toxin, were measured using internal surface reversed‐phase high‐performance liquid chromatography (Fushimi Pharmaceutical Co., Ltd., Kagawa, Japan) (Niwa et al. 1988). IL‐6 levels were analyzed using standard laboratory methods at SRL Inc. (Tokyo, Japan). Interleukin‐1 beta (IL‐1β) and tumor necrosis factor‐α (TNF‐α) levels were determined using enzyme‐linked immunosorbent assay (ELISA) kits (R&D Systems Inc., MN, USA).

### 2.2 Cellular Experiments

#### 2.2.1 Cell Culture

Human umbilical vein endothelial cells (HUVECs) purchased from ScienCell Research Laboratories (Carlsbad, CA, USA) were maintained in endothelial cell medium (ScienCell Research Laboratories) supplemented with 5% fetal bovine serum, 1% endothelial cell growth supplement, and 1% penicillin/streptomycin solution (ScienCell Research Laboratories). Cells were cultured under standard conditions (humidified atmosphere, 5% CO_2_, 37°C) and passaged twice a week.

#### 2.2.2 Preparation of Reagents

IS potassium salt (Nacalai Tesque, Inc., Kyoto, Japan) was dissolved in ultrapure water. Ave (Sigma‐Aldrich, St. Louis, MO, USA) was dissolved in ethanol (Kanto Chemical Co., Inc., Tokyo, Japan). Prazosin hydrochloride (Sigma‐Aldrich), an adrenergic α‐receptor blocker, was dissolved in ultrapure water. Dorsomorphin (DMP) (Fujifilm Wako, Tokyo, Japan), an AMPK inhibitor, was dissolved in dimethyl sulfoxide (DMSO, Fujifilm Wako). The protein kinase B (Akt) inhibitor MK‐2206 2HCl (Selleck Biotech Co., Ltd., Kanagawa, Japan) was dissolved in ultrapure water.

#### 2.2.3 Experimental Protocol

HUVECs were seeded at a density of 1.0 × 10^5^ cells onto 35‐mm dishes (AGC Techno Glass Co., Ltd., Shizuoka, Japan). The following day, the medium was replaced with Dulbecco's modified Eagle's medium (DMEM; Sigma‐Aldrich) supplemented with a 1% penicillin/streptomycin solution and a specific reagent. Inhibitors and blocking reagents were added 1 h before the addition of IS and Ave. After 24 h of IS and Ave treatment, the culture supernatant was collected, and cells were lysed using RNAiso Plus (Takara Bio Inc., Shiga, Japan) for RNA extraction.

#### 2.2.4 Real‐Time qPCR

The concentration of extracted total RNA was measured and adjusted using a NanoDrop One UV‐Vis Spectrophotometer (Thermo Fisher Scientific, Waltham, MA, USA). The RNA was then reverse‐transcribed and amplified using an RT Kit for qPCR and THUNDERBIRD SYBR qPCR Mix (TOYOBO, Osaka, Japan) with specific primer pairs (Table S1) on a QuantStudio 5 real‐time PCR system (Thermo Fisher Scientific). The relative expression levels of target genes were normalized to GAPDH, and data were analyzed using the ΔΔCt method.

#### 2.2.5 Enzyme‐Linked Immunosorbent Assay

The levels of IL‐6 in the culture supernatants were measured using an ELISA. A Human IL‐6 Quantikine ELISA kit (R&D Systems Inc., Minneapolis, MN, USA) was used for the analysis. This assay was performed according to the manufacturer's instructions.

#### 2.2.6 Western Blotting

**FIGURE 1:**
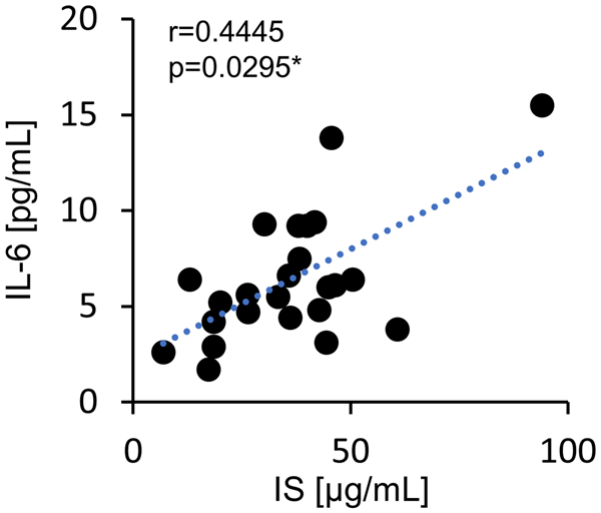
Ave promotes phosphorylation of Akt but is unrelated to anti‐inflammatory effects. (A) Western blotting of cytoplasmic fractions 24 h after adding IS and Ave. Relative quantification of (B) phosphorylated Akt (S473) and (C) Akt under each condition. The β‐actin blot shown here is identical to that in Supplementary Figure 4A. Cytosolic proteins, including phosphorylated Akt (S473), Akt, HO‐1, and β‐actin, were extracted from the same cell culture samples and analyzed together in a single Western blot experiment. Therefore, the β‐actin bands are shared across both panels as internal loading controls for these simultaneously measured targets. (D) IL‐6 concentration in the culture supernatant 24 h after adding the IS and Ave with Akt inhibitor MK‐2206 (MK). All the values are presented as the mean ± SEM, *n =* 6. ** *p <* 0.01, evaluated using the one‐way ANOVA followed by Tukey's post‐hoc test. $$ *p <* 0.01, $ *p <* 0.05, evaluated using the Kruskal‐Wallis, followed by Dunn's post‐hoc test.

HUVECs were cultured in 60‐mm tissue culture dishes at a density of 2.0 × 10^5^ cells per dish. To evaluate the rapid inflammatory signaling, cells were treated with IS and/or Ave for 1 h prior to harvesting for NF‐κB analysis. In contrast, Akt, HO‐1, and nuclear Nrf2 levels were assessed after 24 h of treatment with IS and/or Ave. For Figure 1A and Figure S4A, cytosolic proteins, including phosphorylated Akt (S473), Akt, HO‐1, and β‐actin, were extracted from the same samples and processed in parallel. All target proteins were transferred on the same gel, allowing shared use of β‐actin as a loading control across both figures.

For nuclear and cytoplasmic fractionation, cells were lysed with cytoplasmic lysis buffer (10 mM HEPES [pH 7.9], 10 mM KCl, 1.5 mM MgCl_2_, 1 mM dithiothreitol, 0.5% NP‐40, protease inhibitors, and phosphatase inhibitors) and incubated on ice for at least 15 min. The supernatant was centrifuged at 800 × *g* for 10 min at 4°C to obtain the cytoplasmic fraction. The remaining pellet was resuspended in 100 µL of nuclear extraction buffer (20 mM HEPES [pH 7.9], 400 mM NaCl, 1.5 mM MgCl_2_, 0.2 mM EDTA, 1 mM DTT, 10% glycerol, protease inhibitors, and phosphatase inhibitors). The sample was then sonicated on ice and centrifuged at 14,000 × *g* for 30 min at 4°C to obtain the nuclear fraction. Both fractions were mixed with 4× Laemmli sample buffer for further analysis.

Sodium dodecyl sulfate–polyacrylamide gel electrophoresis was performed on the prepared samples, followed by protein transfer onto Immobilon‐P polyvinylidene fluoride membranes (Merck, Darmstadt, Germany). The target proteins were detected using specific antibodies and visualized with SuperSignal West Femto Maximum Sensitivity Substrate (Thermo Fisher Scientific). Band intensities were analyzed using a Lumino Image Analyzer (ImageQuant LAS 800; Cytiva, Marlborough, MA, USA) and quantified using ImageJ software (version 1.53).

#### 2.2.7 RNA Sequencing

High‐purity RNA was extracted from HUVECs using the ISOSPIN Cell & Tissue RNA Kit (Nippon Gene Co., Ltd., Tokyo, Japan). Complementary DNA was synthesized using the SMART‐Seq v4 Ultra Low RNA‐Seq Kit (Takara Bio Inc.). Library construction was performed using the Nextera XT DNA Sample Preparation Kit (Illumina, Inc., San Diego, CA, USA), and sequencing was performed on an Illumina NovaSeq 6000 v1.5 platform with paired‐end sequencing. FASTQ sequence data were processed on a Linux‐based server. Sequence quality control was performed using FASTP. The human reference genome GRCh38.p14 (GCA_000001405.29) was obtained from the Ensembl database, and sequence reads were mapped to the reference genome using HISAT2. The read counts for each gene were quantified using RSEM. Differentially expressed gene analysis and pathway analyses were conducted using iDEP (Ge et al. 2018) (version 2.0) based on the obtained read count data. Genes exhibiting significantly different expression levels between groups were identified using a false discovery rate threshold of <0.1 and a fold change >2.

#### 2.2.8 High‐Performance Liquid Chromatography

The analysis of Ave incorporation into HUVECs was performed using an Agilent 1200 Series high‐performance liquid chromatography (HPLC) system (Agilent Technologies, Santa Clara, CA, USA) with an Eclipse XDB‐C18 column (4.6 × 150 mm, 5 µm particle size). HUVECs were cultured in 35‐mm tissue culture dishes at a density of 1.0 × 10^5^ cells per dish, and Ave was added after 24 h and collected in PBS 24 h later. Each cell sample was homogenized using a microhomogenizer, followed by the addition of methanol and vortexing. The samples were then centrifuged at 12,000 × *g* at room temperature for 10 min. The supernatants were filtered through a 0.45‐µm filter. The filtered supernatants were concentrated using a centrifugal evaporator at 37°C for 6 h and then reconstituted in 0.05 mL of 80% ethanol. A 0.01‐mL aliquot of the reconstituted sample was injected into the HPLC system under the following conditions, with some modifications based on a previous report (Schär et al. 2018): a flow rate of 1 mL/min with a solvent gradient consisting of solvent A (0.1% formic acid; Fujifilm Wako Pure Chemicals, Osaka, Japan) and solvent B (100% acetonitrile; Kanto Chemical). The gradient program was as follows: 0 min (A:B = 95:5), 30 min (A:B = 80:20), 45 min (A:B = 65:35), 50 min (A:B = 35:65), 60 min (A:B = 0:100), and 61 min (A:B = 95:5). The column temperature was set to 40°C, and spectra were detected using a diode array detector at 325 nm.

### 2.3 Docking Simulation

The structure of AhR was obtained from the Protein Data Bank (PDB ID: 5NJ8, Chain A) (Berman et al. 2000). Because a dedicated single‐structure database for AhR is unavailable, an AhR‐only structure was generated by extracting AhR from the AhR complex using UCSF Chimera (Huang et al. 2014), followed by the removal of unnecessary ligands. The IS and Ave ligands were retrieved from the PubChem database (Kim et al. 2016) in SDF format and converted to the Mol2 format using Open Babel (O'Boyle et al. 2011) (IS: PubChem CID 10258; Ave: PubChem CID 11723200). Hydrogen atoms and atomic charges were added to the AhR structure in UCSF Chimera, and hydrogen atoms were also added to IS and Ave. Docking simulations between the receptor and ligands were performed using the SwissDock web server (Grosdidier et al. 2011), and the results were visualized in UCSF Chimera.

### 2.4 Statistical Analysis

Data are expressed as the mean ± SEM. Statistical analyses were performed using GraphPad Prism version 9.5.1 (GraphPad Software, San Diego, CA, USA). The normality and homogeneity of variance of the data were assessed using the Shapiro‐Wilk test and Bartlett's tests, respectively. If the data followed a normal distribution with equal variance, statistical significance was determined using one‐way analysis of variance, followed by Tukey's post‐hoc test. If the data did not follow a normal distribution or showed unequal variance, statistical significance was determined using the Kruskal‐Wallis test, followed by Dunn's post‐hoc test. The correlation coefficient and p‐value were calculated using Spearman's test. Statistical significance was set at *p <* 0.05.

## 3 Results

### 3.1 Experiment 1: In Hemodialysis Patients, the Concentration of IS in the Serum Correlates With the Concentration of IL‐6 in the Serum

**FIGURE 2:**
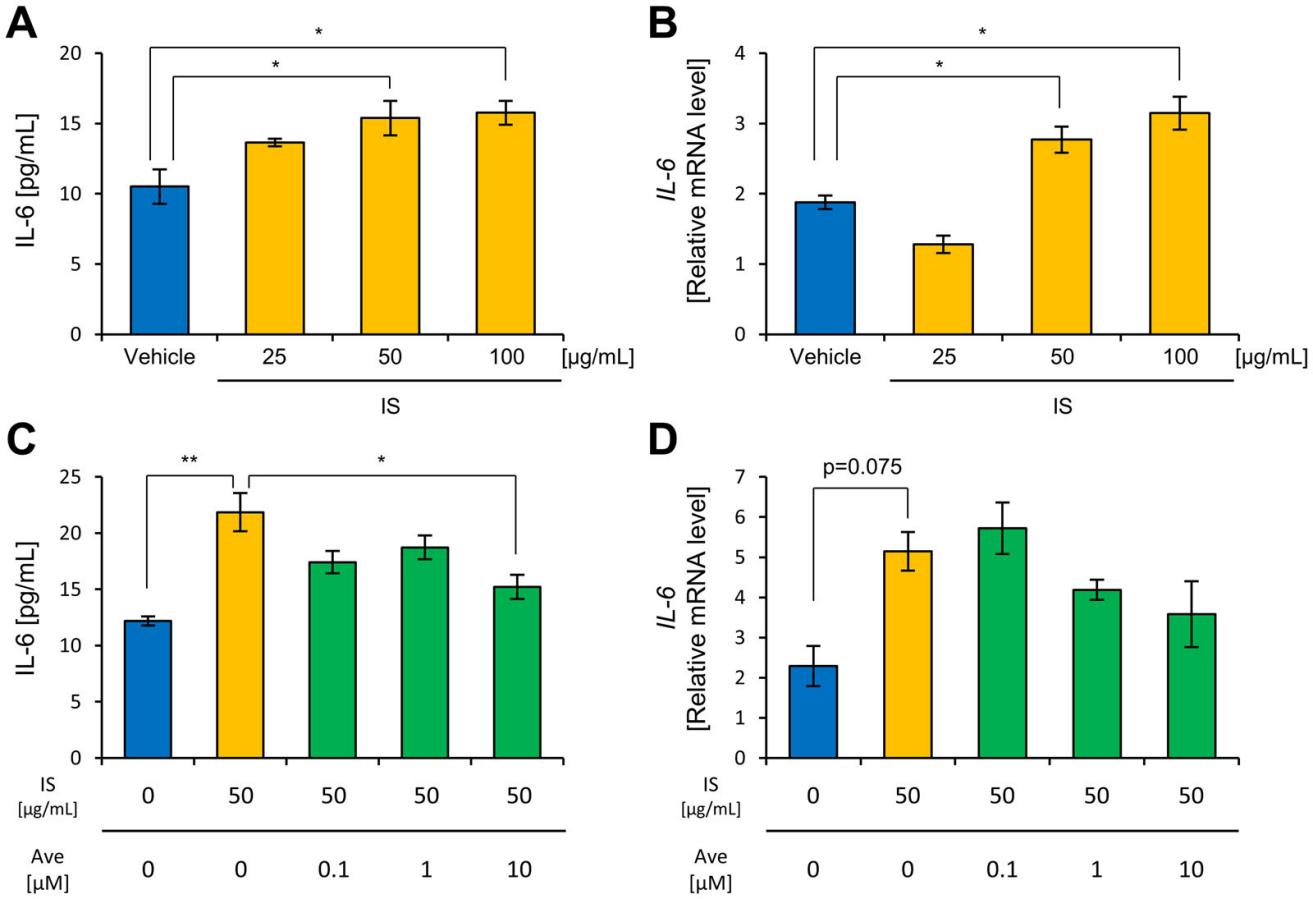
Correlation between serum IS levels and inflammatory cytokines in hemodialysis patients. Scatter plot showing the relationship between serum IS concentration and serum IL‐6 concentration in hemodialysis patients (*n =* 24). The blue dotted line represents the linear approximation curve, and Spearman's correlation coefficient and p‐value are indicated within each plot.

First, we examined the serum concentration of the uremic toxin IS. As a result, a significant correlation was observed only with IL‐6 (Figure 2), whereas no significant correlation was found with the other inflammatory cytokines (IL‐1β, TNF‐α) (Figures S2A and 1B). Therefore, we focused on IL‐6 when investigating the anti‐inflammatory effects of Ave and the underlying mechanisms.

### 3.2 Experiment 2: The Inflammatory Response Is Enhanced by IS at Concentrations of 50 µg/mL or Higher and Suppressed by Ave at Concentrations of 10 µM or Higher

To mimic the conditions of hemodialysis patients as closely as possible, we conducted experiments using IS at concentrations of 25 and 100 µg/mL, with 50 µg/mL as a reference point. Similarly, Ave was tested at concentrations of 0.1, 1, and 10 µM, based on prior studies involving cell experiments.

**FIGURE 3:**
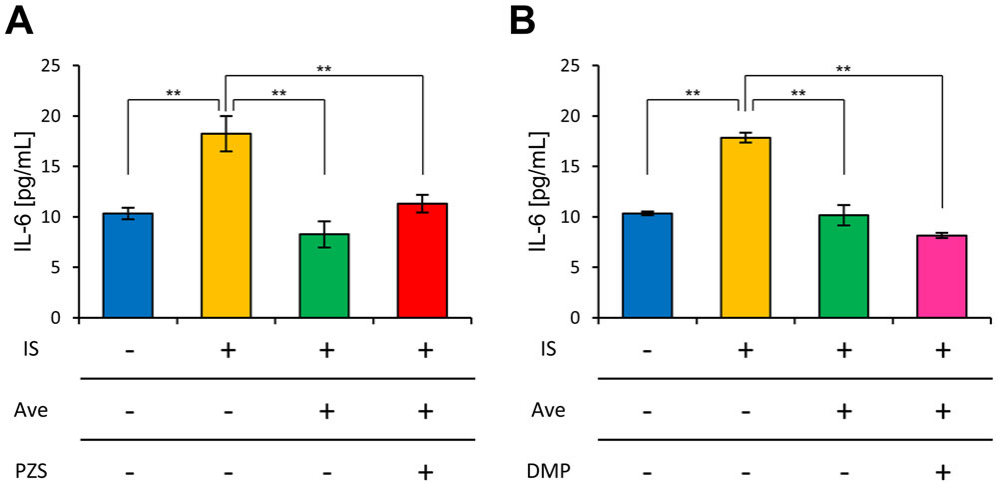
IS increases IL‐6 secretion and gene expression, and treatment with both IS and Ave suppresses the increase in IL‐6. (A) IL‐6 concentration in the culture supernatant 24 h after adding the IS. (B) Gene expression of IL‐6 in HUVECs 24 h after adding the IS. (C) IL‐6 concentration in the culture supernatant 24 h after adding IS and Ave. (D) Gene expression of IL‐6 in HUVECs 24 h after adding IS and Ave. All the values are presented as the mean ± SEM, n = 5. ** *p* < 0.01, * *p* < 0.05, evaluated using the one‐way ANOVA test, followed by Tukey's post‐hoc test.

The amount of IL‐6 secretion and gene expression significantly increased in the groups treated with 50 or 100 µg/mL IS compared with the vehicle group (Figure 3A, B). The group treated with 50 µg/mL IS and 10 µM Ave exhibited significantly lower IL‐6 secretion levels than the group treated with 50 µg/mL IS alone (Figure 3C).

These findings indicate that Ave at a concentration of 10 µM exerts anti‐inflammatory effects in response to IS at 50 µg/mL. Additionally, we investigated other inflammatory markers using 50 µg/mL IS and 10 µM Ave. Since Ave also has antioxidant properties, we examined antioxidant indicators using the same experimental conditions. We observed a decrease in nitric oxide production, suppression of cell adhesion molecule gene expression, and an increase in antioxidant capacity (Figure S2E‐2 M).

### 3.3 Experiment 3: The Anti‐Inflammatory Effect of Ave Is Not Mediated by the Adrenergic α1 Receptor Pathway

Next, we investigated the mechanism underlying the anti‐inflammatory effect of Ave.

**FIGURE 4:**
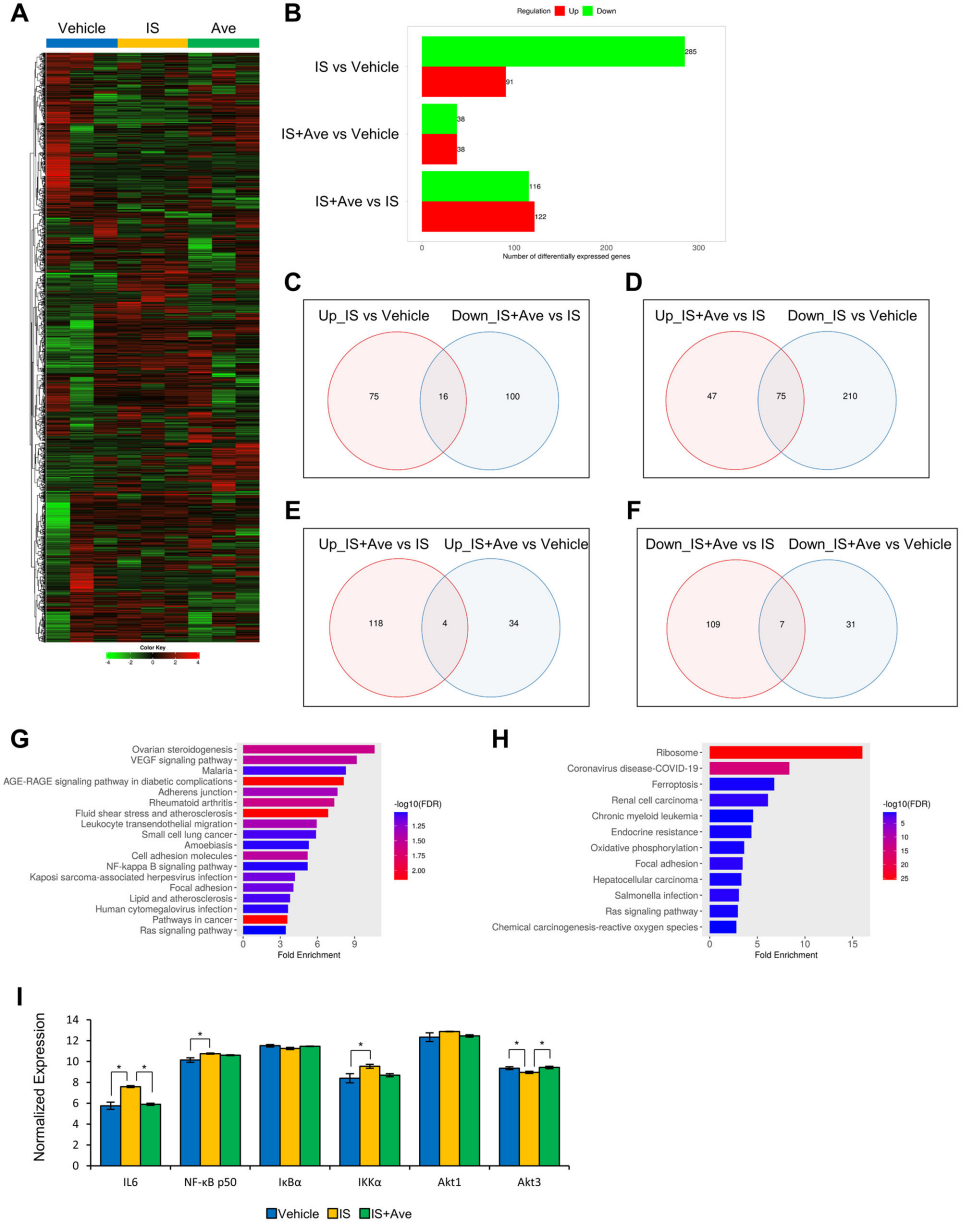
The anti‐inflammatory effect of Ave was not suppressed by the use of adrenergic alpha receptor blockers or AMPK inhibitors. (A) IL‐6 concentration in the culture supernatant 24 h after adding the IS and Ave with adrenergic alpha receptor blocker prazosin (PZS). (B) IL‐6 concentration in the culture supernatant 24 h after adding the IS and Ave with the AMPK inhibitor dorsomorphin (DMP). All the values are presented as the mean ± SEM, *n =* 5. ** *p <* 0.01, evaluated using the one‐way ANOVA, followed by Tukey's post‐hoc test.

When 10 µM Ave was added to 50 µg/mL IS, IL‐6 secretion was significantly suppressed. However, co‐treatment with prazosin, an α1 receptor blocker, did not alter the anti‐inflammatory effects of Ave (Figure 4A).

To examine the involvement of another previously reported pathway, we conducted a similar experiment using dorsomorphin, an AMPK inhibitor. The results showed that Ave's anti‐inflammatory effect was not also suppressed even in the presence of dorsomorphin (Figure 4B).

Based on these findings, we concluded that the anti‐inflammatory effect of Ave is not mediated by the adrenergic α1 receptor pathway.

### 3.4 Experiment 4: The Anti‐Inflammatory Effect of Ave Is Independent of the Akt Signaling Pathway

**FIGURE 5:**
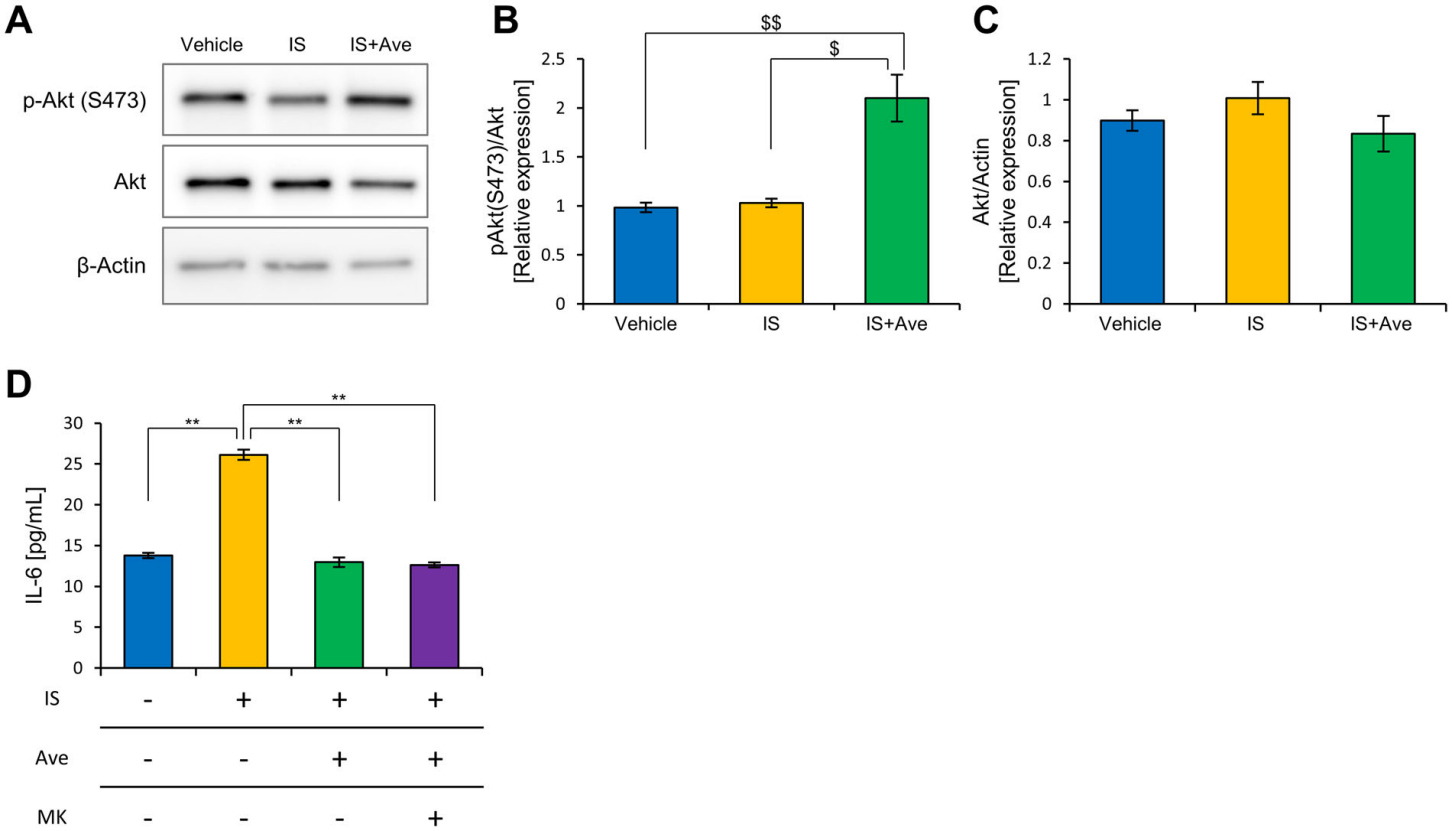
RNA‐seq analysis showed that the addition of IS and Ave to HUVECs may affect the Akt/IKK/IκB signaling pathway. (A) Heatmap of expression patterns of all detected genes. (B) The numbers of differentially expressed genes for each comparison. (C)–(F) Venn diagram shows the number of overlapping genes, ((C): Up_IS vs. Vehicle and Down_IS+Ave vs. IS, (D): Down_IS vs. Vehicle and Up_IS+Ave vs. IS, (E): Up_IS+Ave vs. Vehicle and Up_IS+Ave vs. IS, (F): Down_IS+Ave vs. Vehicle and Down_IS+Ave vs. IS). (G) Pathway analysis results from KEGG gene sets in the pattern (C). (H) Pathway analysis results from using KEGG gene sets in the pattern (D). (I) Expression patterns of genes related to the Akt/IKK/IκB pathway. All the values are presented as the mean ± SEM, *n =* 3. * FDR < 0.1 evaluated using the one‐way ANOVA. To account for multiple tests, p‐values were adjusted using the Benjamini‐Hochberg method, and the False Discovery Rate (FDR) was calculated.

Next, we used RNA‐seq to comprehensively analyze the changes in gene expression following IS and Ave treatment. The gene expression profiles of each group are presented as a heatmap (Figure 5A). Compared with the IS group, 116 genes were downregulated, and 122 genes were upregulated in the IS+Ave group (Figure 5B). To further examine the relationship between the changes in gene expression among the three groups, we analyzed the data using a Venn diagram. Based on previous findings that IS increases IL‐6 secretion and that Ave suppresses this secretion, we generated a Venn diagram for four comparisons (Figure 5C‐F). Next, we performed a pathway analysis. The results for the gene set shown in Figure 5C are presented in Figure 5G, whereas those for the gene set shown in Figure 5D are presented in Figure 5H. Based on the Kyoto Encyclopedia of Genes and Genomes pathway database, IL‐6 was found to be involved in several signaling pathways, including the AGE‐RAGE signaling pathway, rheumatoid arthritis, amoebiasis, Kaposi sarcoma‐associated herpesvirus infection, lipid and atherosclerosis, human cytomegalovirus infection, cancer, coronavirus diseases (COVID‐19), and Salmonella infection (Figures 5G, H). In many of these pathways, NF‐κB was identified as an upstream regulator. Therefore, we decided to focus on NF‐κB‐related pathways for further analysis. When we examined the NF‐κB signaling pathway in the Kyoto Encyclopedia of Genes and Genomes database, we found that the gene expression levels of NF‐κB p50, inhibitor of nuclear factor kappa B (IκBα), and IκB kinase α (IKKα) were altered (Figure S3). Additionally, the Akt/IKK/IκB pathway was identified as an upstream regulator of NF‐κB, prompting us to analyze the expression of genes related to this pathway (Figure 5I). These findings suggested that the Akt/IKK/IκB signaling pathway may play a role in the anti‐inflammatory mechanism of Ave.

We performed western blotting to measure Akt phosphorylation in the cytoplasm. Contrary to the hypothesis established by RNA‐seq, Akt phosphorylation was enhanced only in the IS+Ave group (Figure 1A–C). These results suggest that the anti‐inflammatory effect of Ave may be associated with the activation of Akt signaling through the promotion of Akt phosphorylation. Herein, we investigated the antioxidant and nuclear factor erythroid 2‐related factor 2 (Nrf2) pathways as potential downstream pathways of Akt phosphorylation that may contribute to the anti‐inflammatory effects of Ave. To assess these pathways, we measured heme oxygenase 1 levels in the cytoplasm and Nrf2 levels in the nucleus using western blotting. Both were significantly reduced in the IS+Ave group, suggesting that the anti‐inflammatory effects of Ave are not mediated by antioxidant mechanisms or Nrf2‐dependent pathways (Figure S4A–D).

Next, we investigated whether the anti‐inflammatory effects of Ave were suppressed by MK‐2206, an Akt inhibitor. IL‐6 secretion remained significantly reduced in the IS+Ave group, even after MK‐2206 treatment, compared with that in the IS group (Figure 1D).

### 3.5 Experiment 5: Ave Is Incorporated Into HUVECs and May Modulate AhR Signaling, Exerting Anti‐Inflammatory Effects

We hypothesized that Ave competitively inhibited IS binding to AhR, thereby suppressing the IS‐induced inflammatory response. To explore this possibility, we performed in silico docking simulations to evaluate the potential interactions between IS or Ave and AhR.

**FIGURE 6:**
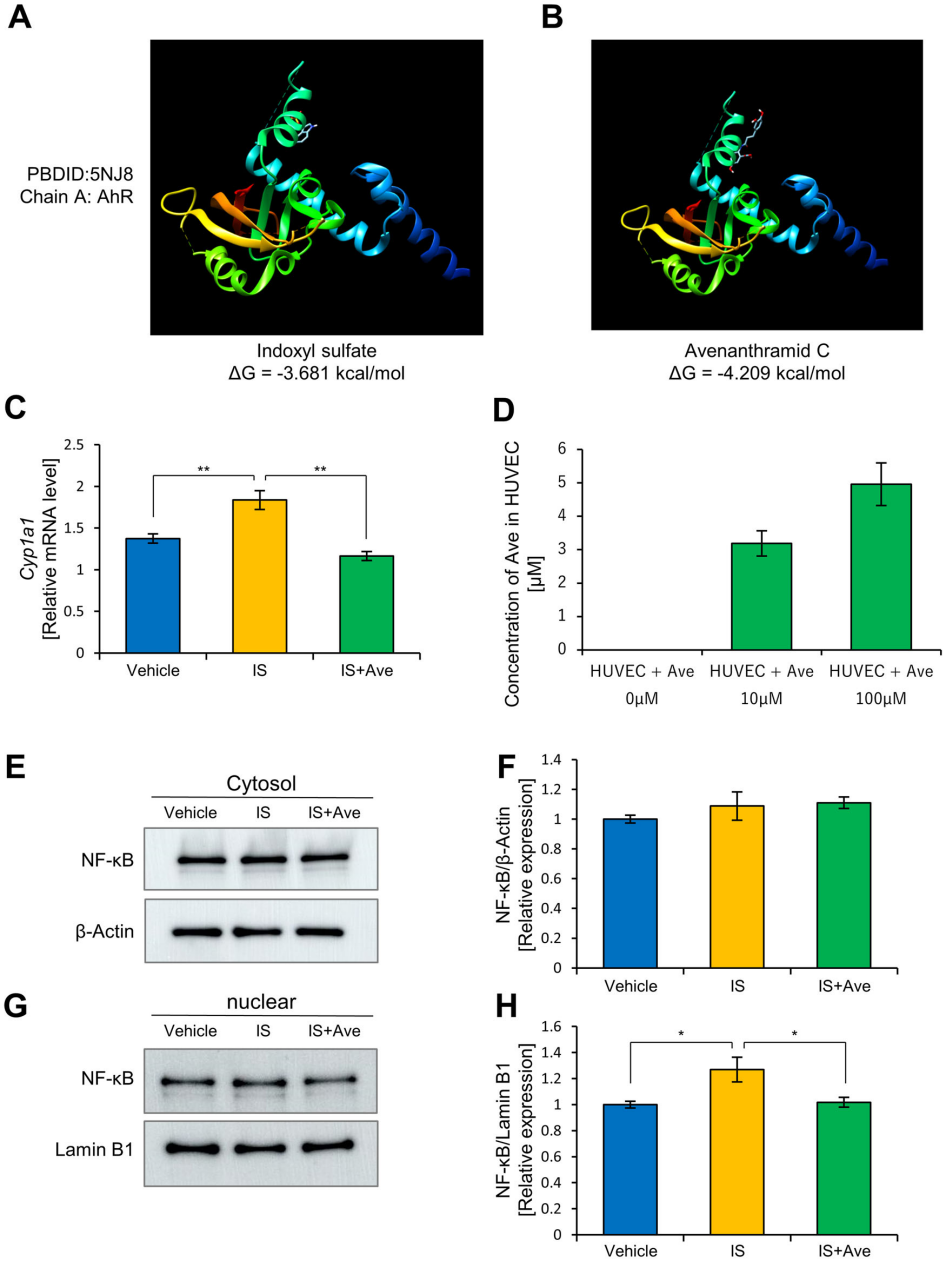
Ave inhibits AhR and suppresses *Cyp1a1* gene expression in HUVECs by competitive inhibition. The predicted docking positions of (A) IS (PubChem CID: 10258) and (B) Ave (PubChem CID: 11723200) with AhR (PDB ID: 5NJ8; Chain A). The docking simulations were performed using the AutoDock Vina tool on the SwissDock platform. (C) Gene expression of *Cyp1a1* in HUVECs 24 h after adding the IS and Ave. The (C) values are presented as the mean ± SEM, *n =* 5. ** *p <* 0.01, evaluated using the one‐way ANOVA, followed by Tukey's post‐hoc test. (D) Quantification of Ave incorporated into HUVECs. The (D) values are presented as the mean ± SEM, *n =* 3. (E) Western blot of cytoplasmic fractions at 1 h after adding IS and Ave. (F) Relative quantification of NF‐κB in the cytoplasmic. (G) Western blot of nuclear fractions at 1 h after adding IS and Ave. (H) Relative quantification of NF‐κB in nuclear fraction. The values in (F) and (H) are presented as mean ± SEM (Vehicle: *n =* 4, IS: *n =* 5, IS+Ave: *n =* 5). * *p <* 0.05, evaluated using the one‐way ANOVA followed by Tukey's post‐hoc test.

Chain A of PDBID: 5NJ8 was obtained from the Protein Data Bank. Because the ligands were bound to leucine at residue 112 and glutamic acid at residue 116 in Chain A, we performed docking simulations of IS and Ave targeting these two residues (Figure 6A–B). The binding free energy of the obtained models was −3.681 kcal/mol for IS and −4.209 kcal/mol for Ave, indicating that Ave had a stronger binding affinity. Additionally, when we examined the details of the binding interactions, the distance between glutamic acid 116 and the indole group of IS was 3.49 Å, suggesting the potential formation of a hydrogen bond between the nitrogen of the indole group and the carboxyl group of glutamic acid. The distance between leucine 112 and the C‐chain of the amide bond in Ave was 3.138 Å, suggesting a possible hydrophobic interaction between the aromatic ring of Ave and the isobutyl group of leucine. Furthermore, Ave was docked in a manner that covered glutamic acid 116, raising the possibility that Ave inhibited IS binding.

We next measured the expression of cytochrome P450 family 1 subfamily A member 1, an AhR target gene. The results showed that cytochrome P450 family 1 subfamily A member 1 expression, which was significantly upregulated in the IS group compared with that in the vehicle group, was significantly suppressed in the IS+Ave group (Figure 6C).

If Ave binds to the AhR receptor in cells, it must first enter the HUVECs. To confirm this, HUVECs were treated with Ave 10 and 100 µM for 24 h; the cells’ contents were extracted and analyzed using HPLC. The chromatographic analysis confirmed that Ave was incorporated into cells (Figure 6D, Figure S5).

To further examine the mechanism underlying IL‐6 upregulation, we analyzed the localization of NF‐κB in cytoplasmic and nuclear fractions of HUVECs by western blotting. Treatment with 50 µg/mL IS significantly increased the nuclear translocation of NF‐κB compared with the vehicle group, while co‐treatment with 10 µM Ave significantly attenuated this IS‐induced nuclear accumulation of NF‐κB. In contrast, NF‐κB levels in the cytoplasmic fraction remained unchanged across all groups (Figure 6E‐6H). These results suggest that Ave suppresses IS‐induced IL‐6 secretion, at least in part, by inhibiting the nuclear translocation and activation of NF‐κB.

## 4 Discussion

In this study, we identified the following key findings: 1) IS appeared to be particularly associated with inflammatory responses mediated by IL‐6 both in CKD patients and cultured endothelial cells, 2) Ave suppressed IS‐induced inflammatory responses, and 3) Ave may modulate AhR signaling, which could contribute to the suppression of the inflammatory response.

Our previous studies have shown that the blood concentration of IS in hemodialysis patients is approximately 50 µg/mL (Nagasawa et al. 2024; Nagasawa et al. 2021). Additionally, other studies have also reported that the average blood IS concentration in 234 patients with CKD was 45.3 µg/mL (Lin et al. 2020), while in 31 hemodialysis patients, it fluctuated between 48.56 and 54.36 µg/mL (Wu et al. 2024). In this study, these findings indicated that our in vitro model successfully mimicked the inflammatory response observed in hemodialysis patients. The Ave concentration used in this study was based on in vitro experiments demonstrating its antioxidant effects at 1 µM rather than on its pharmacokinetics in human blood (Serreli et al. 2021). Therefore, the actual human physiological environment may differ significantly from the in vitro conditions in terms of the administered Ave concentration. The average daily Ave intake from oats in humans has been reported to range from 0.3 to 2.1 mg, with a bioavailability of only 0.16% to 2.71% (Tosh and Bordenave 2020). Additionally, one study reported that when individuals consumed oat cookies containing 7.85 mg, the Ave concentration in their blood ranged from 3 to 6 ng/mL (Zhang et al. 2017). The Ave concentration at which anti‐inflammatory effects were observed in this study, 10 µM, is estimated to be approximately 3.15 µg/mL. Therefore, in human trials, significantly higher Ave intake may be required to achieve similar anti‐inflammatory effects. Additionally, we collected culture supernatants and cells 24 h after IS and Ave treatment to assess the inflammatory response. However, when Ave is consumed orally, its blood concentration peaks within 2–3 h, and it is metabolized within 10 h (Zhang et al. 2017). This indicates that the duration of Ave's action differs substantially between human physiological environments and in vitro conditions. Despite substantial differences between human physiological conditions and in vitro environments, previous studies have reported that the antioxidant effect of Ave was observed at a blood concentration of 89.0 nmol/L (Chen et al. 2007) and that antioxidant and anti‐inflammatory effects were achieved with the consumption of 9.2 mg of Ave (Koenig et al. 2014). Therefore, future human trials involving hemodialysis patients, in which the intake of oats and Ave is measured along with the corresponding blood Ave concentration, may help determine the optimal intake of oats and Ave required to achieve anti‐inflammatory effects in humans.

A previous study reported that Ave acts on the adrenergic α1 receptor, activates AMPK, and suppresses IL‐6 secretion (Ramasamy et al. 2020). We examined whether the anti‐inflammatory effects of Ave would be suppressed by prazosin, an α1‐adrenergic receptor blocker, or dorsomorphin, an AMPK inhibitor; however, neither inhibitor affected the anti‐inflammatory effects of Ave. Therefore, we further explored its mechanism of action and considered the possibility that Ave suppresses the IS‐induced inflammatory response by modulating AhR/NF‐κB signaling. While no previous studies have examined the interaction between Ave and AhR, our findings may suggest a potential role for AhR modulation in Ave's mechanism of action. A broad range of polyphenols have been suggested to function as AhR antagonists and may play a role in the prevention and treatment of various diseases and disorders (Xue et al. 2017). Resveratrol, which is abundant in red grape skin, has long been recognized as an AhR antagonist (Whitlock and Baek 2012). Furthermore, a study using HUVECs has reported that resveratrol acts as an AhR antagonist in the presence of 3‐methylcholanthrene, an AhR agonist (Pang et al. 2008). Additionally, chrysin, apigenin, naringenin, and quercetin have been shown to inhibit AhR activity (Santana et al. 2024). However, it has also been suggested that, although polyphenols act as AhR antagonists, they may exert antioxidant and anti‐inflammatory effects through AhR‐independent mechanisms, indicating that the full spectrum of their biological activities cannot be explained solely by AhR modulation (Brinkmann et al. 2022). Other potential mechanisms of action of Ave include the activation of the Nrf2/ARE signaling pathway (Ma et al. 2023), the direct inhibition of NF‐κB phosphorylation by Ave (Wang and Eskiw 2019), and the PI3K/Akt/Nrf2/glycogen synthase kinase‐3 beta pathway (Wankhede et al. 2023). Previous research on the α1‐adrenergic receptor has been conducted using an Alzheimer's disease model with hippocampal neurons as the target, and the reported mechanism of action was identified in ex vivo experiments using hippocampal slices (Ramasamy et al. 2020). In another study, an in vitro model of childhood pneumonia has demonstrated that Ave reduced reactive oxygen species‐induced mitochondrial damage and suppressed inflammatory responses by inducing PI3K/Akt phosphorylation (Pu et al. 2022). Additionally, in a study using human lung epithelial A549 cells, Ave suppressed hypoxia‐induced cyclooxygenase‐2 expression and reduced lung inflammation via sirtuin 1 activation (Lim and Kang 2020). Moreover, in a study using the HUVEC‐derived cell line EA.hy926, where vascular endothelial dysfunction was induced by H_2_O_2_, Ave was found to activate the Nrf2/heme oxygenase 1 pathway, suppress NF‐κB activity, and contribute to vascular function improvement (Baik et al. 2025). As described above, Ave's mechanism of action may vary significantly depending on the target organ, cell type, and experimental conditions. The findings of this study represent only one aspect of Ave's mechanism of action, and it is possible that an HUVEC‐specific pathway exists. Taken together, these findings suggest that modulation of AhR signaling may represent one of the mechanisms underlying the anti‐inflammatory effects of Ave. However, given the complexity of polyphenol–AhR interactions and the involvement of multiple signaling pathways, AhR modulation is likely to be one of several complementary mechanisms contributing to Ave's biological activity. Other Aves have also been reported to exhibit biological effects. AveA has been shown to suppress colorectal cancer growth via the microRNA‐129‐3p/p53‐induced protein with a RING‐H2 domain/p53 signaling pathway (Fu et al. 2019), while AveB has been reported to improve intestinal barrier function by inhibiting the Toll‐like receptor 4/NF‐κB signaling pathway (Liu et al. 2024). Although molecular docking and suppression of AhR target genes suggest that Ave may modulate AhR signaling, further studies are needed to confirm its direct binding and antagonist activity.

Techniques such as bio‐layer interferometry (BLI) or isothermal titration calorimetry (ITC) could be employed in future research to quantify the binding affinity of Ave to AhR. However, these approaches require high‐cost instrumentation or outsourcing, which are not feasible in our current research setting.

Nonetheless, our results provide multiple lines of indirect but functionally relevant evidence. Specifically, Ave suppressed the IS‐induced expression of *Cyp1a1*, a canonical AhR target gene, and also inhibited NF‐κB nuclear translocation, which is considered downstream of AhR activation. Furthermore, docking simulations predicted a stable interaction between Ave and the AhR ligand‐binding domain. Taken together, these findings support the possibility that Ave may modulate AhR signaling, although further biochemical or biophysical validation will be necessary.

The following limitations exist in this study. The number of subjects was only 24, and the clinical trial was limited to a limited geographical area. Recruiting more subjects is necessary to clarify the relationship between IS and IL‐6. Furthermore, since only IL‐6 showed a correlation with IS in the clinical trial in this study, we focused solely on the IL‐6 pathway for in vitro investigations. However, the relationship between other inflammatory markers such as TNFα and IL‐1β and IS requires further detailed examination of large clinical trials and in vitro tests using HUVECs.

In summary, Ave suppresses IS‐induced inflammation and reduces IL‐6 secretion in HUVECs. Although further studies are required to confirm direct interaction with AhR/NF‐κB signaling, Ave shows potential as a functional food component for reducing vascular inflammation in CKD.

## Author Contributions

**Hiroyuki Sasaki**: methodology, formal analysis, investigation, writing – original draft. **Hirofumi Masutomi**: conceptualization, methodology, formal analysis, investigation, writing – original draft. **Hajime Nagasawa**: conceptualization, methodology, formal analysis, investigation, writing – review and editing. **Yuma Matsumoto**: methodology, investigation. **Teruyuki Okuma**: investigation, writing – review and editing. **Tomoyuki Otsuka**: writing – review and editing. **Katsuyuki Ishihara**: conceptualization, writing – review and editing, supervision. **Yusuke Suzuki**: writing – review and editing. **Seiji Ueda**: conceptualization, methodology, writing – review and editing, supervision.

## Conflicts of Interest

Hiroyuki Sasaki, Hirofumi Masutomi, and Katsuyuki Ishihara are employees of Calbee Inc. The remaining authors declare no conflicts of interest.

## Supporting information




**Supplementary Table**: jfds70841‐sup‐0001‐TableS1.docx

**Supplementary Figures**: jfds70841‐sup‐0002‐Figure.docx
