# Supplementary material for: Avenanthramide C From Oats Possibly Exerts Anti‐Inflammatory Effects in Human Umbilical Vein Endothelial Cells
Source: J Food Sci. 2026 Jan 9;91(1):e70841. doi: 10.1111/1750-3841.70841 (PMC12785505; doi:10.1111/1750-3841.70841)
Supplement: Supplementary file 1 — Supplementary Table: jfds70841‐sup‐0001‐TableS1.docx [file JFDS-91-0-s001.docx]

Supplemental Table 1. Primer sequences for RT-qPCR analysis

| *Gapdh* | 5′-AGGGTGGTGGACCTCAT-3′ |
| --- | --- |
|  | 5′-TGAGTGTGGCAGGGACT-3′ |
| *IL-6* | 5′-TTCTCCACAAGCGCCTTCGGTCCA-3′ |
|  | 5′-AGGGCTGAGATGCCGTCGAGGATGTA-3′ |
| *NOS2* | 5′-CACCATCCTCTTTGCGACA-3′ |
|  | 5′-GCAGCTCAGCCTGTACT-3′ |
| *MCP-1* | 5′-TCTCAGTGCAGAGGCTCGCGA-3′ |
|  | 5′-GAGTGAGTGTTCAAGTCTTCG-3′ |
| *ICAM-1* | 5′-TTGGGCATAGAGACCCCGTT-3′ |
|  | 5′-GCACATTGCTCAGTTCATACACC-3′ |
| *VCAM-1* | 5′-TTTGACAGGCTGGAGATAGACT-3′ |
|  | 5′-TCAATGTGTAATTTAGCTCGGCA-3′ |
| *NRF2* | 5′-GACGGTATGCAACAGGACAT-3′ |
|  | 5′-TGGCTTCTGGACTTGGAAC-3′ |
| *SOD-1* | 5′-CCTCGGAACCAGGACCT-3′ |
|  | 5′-TTAATGCTTCCCCACACCTT-3′ |
| *SOD-2* | 5′-GGTTGGCTTGGTTTCAATAAGG-3′ |
|  | 5′-TGCTCCCACACATCAATCC-3′ |
| *HO-1* | 5′-AAAGGAGGAAGGAGCCTATGG-3′ |
|  | 5′-TCAATGTGTAATTTAGCTCGGCA-3′ |
| *CYP1A1* | 5′-CTCAGTACCTCAGCACGCAC-3′ |
|  | 5′-CGTGGCCGACATGGAGATT-3′ |
