# Supplementary material for: Avenanthramide C From Oats Possibly Exerts Anti‐Inflammatory Effects in Human Umbilical Vein Endothelial Cells
Source: J Food Sci. 2026 Jan 9;91(1):e70841. doi: 10.1111/1750-3841.70841 (PMC12785505; doi:10.1111/1750-3841.70841)
Supplement: Supplementary file 2 — Supplementary Figures: jfds70841‐sup‐0002‐Figure.docx [file JFDS-91-0-s002.docx]

**Supplemental Figure 1. Correlation between serum IS levels and inflammatory cytokines in hemodialysis patients.**

Scatter plot showing the relationship between serum IS concentration and serum (A) IL-1β, and (B) TNF-α concentration in hemodialysis patients (n = 24). The blue dotted line represents the linear approximation curve, and Spearman's correlation coefficient and p-value are indicated within each plot.

**Supplemental Figure 2. Various factors related to inflammation and oxidation are enhanced by IS, and Ave suppresses.**

Concentration of (A) Nitric oxide, and gene expression of (B) NOS2, (C) MCP-1, (D) ICAM-1, (E) VCAM-1, (F) NRF2, (G) SOD-1, (H) SOD-2, (I) HO-1 in HUVECs 24 hours after adding the IS and Ave. All the values are presented as mean ± SEM (Vehicle: n=5, IS: n=5, IS+Ave: n=5, LPS: n=5). * p< 0.05, evaluated using the one-way ANOVA followed by Tukey’s post-hoc test. $ p<0.05, evaluated using the Kruskal-Wallis followed by Dunn’s post-hoc test.


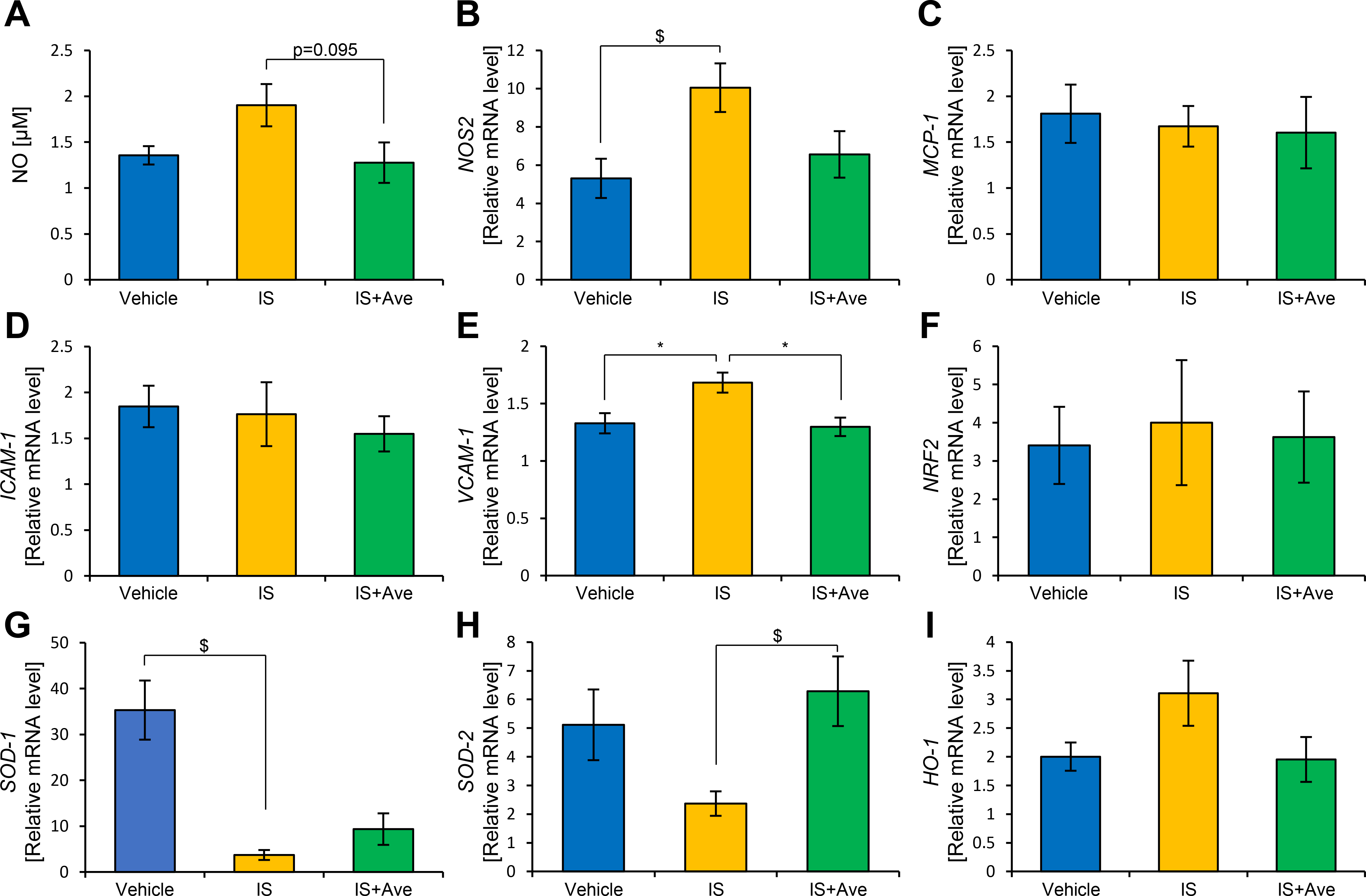


**Supplemental Figure 3. The NF-κB signaling pathway is activated by IS.**

Expression profiles of NF-kappa B signaling pathway related genes visualized on a KEGG pathway diagram using the Pathview package. Red and green indicate genes induces or suppressed by IS, respectively.

**Supplemental Figure 4. IS and Ave decreased HO-1 in the cytoplasm and decreased Nrf2 in the nucleus.**

(A) Western blot of cytoplasmic fractions at 24 hours after adding IS and Ave. (B) Relative quantification of HO-1. (C) Western blot of nuclear fractions at 24 hours after adding IS and Ave. (D) Relative quantification of Nrf2. All the values are presented as mean ± SEM (Vehicle: n=6, IS: n=6, IS+Ave: n=6). ** p< 0.01, * p<0.05, evaluated using the one-way ANOVA followed by Tukey’s post-hoc test. $$ p<0.01, evaluated using the Kruskal-Wallis followed by Dunn’s post-hoc test.

**Supplemental Figure 5. Ave administered to HUVECs may be incorporated into the cells after 24 hours.**

Representative chromatograms showing the measurement of 20 µM Ave using high-performance liquid chromatography, as well as chromatograms of untreated HUVECs and HUVECs treated with Ave at concentrations of 10 µM and 100 µM. The retention time (RT) and peak area values are shown in the figure.
